# Management of Computerized Cognitive Training Programs in Children with ADHD: The Effective Role of Decision Support Systems

**Published:** 2018-10

**Authors:** Marjan GHAZISAEEDI, Leila SHAHMORADI, Sharareh R. NIAKAN KALHORI, Azadeh BASHIRI

**Affiliations:** Dept. of Health Information Management, School of Allied Medical Sciences, Tehran University of Medical Sciences, Tehran, Iran

## Dear Editor-in-Chief

Attention-Deficit Hyperactivity Disorder (ADHD) as a most common, impairing and complex Neuro-behavioral disorder, is characterized by deficits in behavioral features including inattention, too much activity and impulsiveness that affect daily life of children. This mental health condition is diagnosed based on clinical evaluation of such symptoms in children. Untreated children can be faced with a number of difficulties in educational, social and other aspects of life, especially in their future. Therefore, the ideal rehabilitation of children with ADHD can improve the quality of their life and prevent many anomalies in adulthood. Previous studies and clinical experiences have proven the combination of behavioral and pharmacological interventions in the effective rehabilitation of children with ADHD (1, 2). Computerized cognitive training programs like behavioral-mental gyms provide a wide range of cognitive exercises. These programs are provided with regard to the neuro-plasticity of brain that can repair some of its injuries by using special and continuous exercises. Cognitive training programs have quick feedback and unlike drug treatments do not have any side effects (2, 3).

The impairment in cognitive skills such as attention is a common symptom in children with ADHD. Therefore, computerized cognitive training programs as supplementary interventions can provide promising results in the rehabilitation of them (3). Each computerized cognitive training program produces a wide range of games to improve the different type of cognitive skills such as attention, focus, working memory, response control, processing speed, conceptual reasoning, visual perception and other skills in psychological disorders. Brain train, Lumosity, Cogmed, CogniFit are the only examples of the huge volume of these programs. For example, Table 1 shows the games delivered by Captain’s log in the rehabilitation of general attention, divided attention, alternating attention, selective attention, sustain attention, focused attention, response control, working memory and visual perception in the children with ADHD (2–4). Along with the developing extensive volume of computerized cognitive programs in improving psychological conditions such as ADHD, clinicians want to discriminate and select the best programs to improve a special skill or several skills in the patients. In these situations, it seems necessary to develop clinical decision support systems (CDSS). These systems are designed to assist clinicians to make decisions in real time and select the best option of treatments. CDSS can individualize treatment methods according to the patient’s situation and guarantees an algorithmic approach to the treatment of patients (5, 6).

**Table 1:** The games delivered by Captain’s log in the rehabilitation of some variables as for children with ADHD

**Cognitive skills**	**Caption**’s **log (Games)**
General Attention	The Great Hun, Match Point, Cat’s Play, Watchdog, Red Light, Green Light, Total Recall, Tower Power, Max’s Match, What’s Next, Conceptor, Figure It Out, What’s Missing, Bits And Pieces, City Lights, Counting Critters, Great Escape, Darts!, On The Road, Code Cracker, Tricky Tracks, Puzzle Power, Remember The, Alamo, Match Play, Racing Robots, Bingo Discovery, Eureka, Touchdown, A Day At The Races?, Where’s My Car, Birds Of A Feather, Lost And Found, Don’t Be Late, ?, Forget Something
Divided Attention	Mystery Messages, On The Road, Hide And Seek
Alternating Attention	Mouse Hunt, Red Light, Green Light, Match Maker, Great Escape
Selective Attention	Smart Detective, Target Practice, Happy Trails, Match Maker
Sustain Attention	Target Practice, Mouse Hunt, Pop-N-Zap
Focused Attention	Drum Signals, Musical Pairs, Target Practice, Domino Dynamite, Eagle Eye, Pick And Pop, Happy Hunter, Pop-N-Zap
Response control	Darts!, Cat’s Play, Mouse Hunt, Red Light, Green Light
	Target Practice, Pick Quick, Match Point
Working memory	Mystery Messages, Smart Detective, Drum Signals, Musical Pairs, The Ugly Duckling, Happy Trails, Total Recall, Domino Dynamite, What’s Next, Bits and Pieces, City Lights, Counting Critters, Happy Hunter
Visual Perception	Cat’s Play, Target Practice, The Ugly Duckling, Domino Dynamite, Tower Power, What’s Next, Conceptor, Eagle Eye, Pick and Pop, Figure it Out, Bits and Pieces, Match Maker, Great Escape, Pick Quick, Pop-N-Zap, Darts!, On the Road, Hide and Seek, Concentration

The role of clinical decision support systems in the management of rehabilitation of children with ADHD is very effective and future studies should pay attention to this issue.
